# Spatiotemporal regulation of enhancers during cardiogenesis

**DOI:** 10.1007/s00018-016-2322-y

**Published:** 2016-08-06

**Authors:** Laurent Dupays, Timothy Mohun

**Affiliations:** The Francis Crick Institute, Mill Hill Laboratory, The Ridgeway, Mill Hill, London, NW7 1AA UK

**Keywords:** Transcription, Heart, Development, Embryonic stem cell

## Abstract

With the advance in chromatin immunoprecipitation followed by high-throughput sequencing, there has been a dramatic increase in our understanding of distal enhancer function. In the developing heart, the identification and characterisation of such enhancers have deepened our knowledge of the mechanisms of transcriptional regulation that drives cardiac differentiation. With next-generation sequencing techniques becoming widely accessible, the quantity of data describing the genome-wide distribution of cardiac-specific transcription factor and chromatin modifiers has rapidly increased and it is now becoming clear that the usage of enhancers is highly dynamic and complex, both during the development and in the adult. The identification of those enhancers has revealed new insights into the transcriptional mechanisms of how tissue-specific gene expression patterns are established, maintained, and change dynamically during development and upon physiological stress.

## Introduction

The development of the four-chambered heart involves coordinate differentiation of multiple cardiac cell types, such changes occurring, whilst the embryonic organ maintains its essential function pumping blood through the developing embryo [1–3]. The transition between different differentiation states of cardiac progenitors during heart morphogenesis is the result of a tightly coordinated process, regulated at the transcriptional level. In that context, the non-coding part of the genome has recently been shown to encode a large collection of enhancers and associated non-coding RNAs (for example micro RNAs or long non-coding RNAs) with regulatory functions within the gene regulatory network driving heart development [4, 5]. Distant enhancers control the dynamic expression of cardiac-specific genes and any disruption of such controls can lead to a large variety of congenital heart defects (CHD). CHD are the most common type of birth defect, affecting 10 % live births [6, 7]. Moreover, mutations within non-coding elements, such as enhancers, have been shown to disrupt heart development [8–10]. Our increased understanding of the transcriptional processes occurring during cardiac development has spurred efforts to identify the mechanisms that would allow the generation of cardiomyocytes in vitro in numbers large enough for therapeutic use. Efforts have focused either on differentiating embryonic stem cells, or using induced pluripotent stem cells, terminally differentiated cells which have been reprogrammed through the introduction of a defined set of genes [11–13].

After describing the common strategies used to identify cardiac enhancers and the difficulty in the design and interpretation of ChIP-seq experiments, we will summarise the state of our knowledge on the dynamics of enhancer usage during the cardiac development and differentiation.

## Identification of cardiac enhancers

Gene expression is regulated through the integrated action of different types of cis-regulatory elements, such as distal enhancers [14, 15]. Classically, enhancers are defined as non-coding DNA elements that can increase the transcription of genes located in cis. With a significant role in regulating gene expression, the identification and location in the genome of those enhancers have been the focus of many studies in the cardiovascular development [16].

However, by their nature, the identification of such enhancers is challenging. For example, their location relative to the target gene, or genes, is highly variable and they can function in an orientation-independent manner. Prior to the development of high-throughput sequencing methods, enhancers were largely identified by comparative genomics, with the assumption that non-coding sequence which is conserved between different vertebrate or mammalian species is enriched for enhancers [17, 18]. Using this method in combination with functional assays, it was found that about half of the identified, highly conserved sequences are, indeed, functionally active enhancers. However, this approach has some important limitations. Some conserved sequences have no apparent enhancer function when assayed in transgenic mice. Moreover, it is now clear that a substantial fraction of cardiac enhancers displays modest or no conservation across species [19–22].

With advances in high-throughput sequencing techniques, new approaches have been developed to identify gene enhancers [23]. Two types of strategies have been used to identify cardiac enhancers: genome-wide distribution of key cardiac transcription factors and the identification of appropriate epigenomic marks [16]. Each strategy utilises ChIP-seq (chromatin immunoprecipitation followed by high-throughput sequencing) and is independent of cross-species sequence conservation. Both have proved powerful in identifying cardiac enhancers, but both have important limitations that we will summarise.

## Experimental design and data analysis challenges

The genome-wide distribution of post-translational histone modifications of chromatin, (or “chromatin marks”) has widely been used to predict enhancer activity [24]. The identification of de novo mutations in histone-modifying genes in CHD emphasizes the importance of chromatin modification in cardiac differentiation [25]. The protocols used to identify such marks are now well established and multiple different marks have been identified, for example, H3K4me1 correlates with gene promoters, H3K4me3 with active promoters, H3k27me3 with inactive promoters, and H3K27ac with some active enhancers. Importantly, there is no single chromatin mark which can be used to identify all active enhancers. Furthermore, new histone modifications correlating with the presence of new classes of active enhancers are identified regularly [26].

Another difficulty with this approach is the impact of tissue contamination when ChIP-seq is undertaken with cardiac tissue or with the whole organ. The heart contains a large number of different cell types, (cardiomyocytes, fibroblasts, endothelial and smooth muscle cells, and to name a few) and their precise relative contributions have been the subject of numerous debates [27]. Studies of chromatin marks using cardiac tissue will, therefore, identify an assortment of enhancer activities reflecting the contribution of multiple cell types. Single-cell technology offers a potential answer, but there are obvious technical difficulties with the use of such small quantities of starting material and no such study has yet been reported for cardiac cells [28, 29]. Indeed, one limitation of the ChIP-seq technique is the relatively large amount of initial material required, due to low cellular DNA recovery rate. In a single-cell experiment, non-specific binding of the antibody during the immunoprecipitation step leads to experimental noise. New methods are currently being developed to address this specific problem [30]. For instance, microfluidic technologies have been developed to isolate single cells into aqueous droplets, in which the chromatin of individual cells is labelled with a unique oligonucleotide barcode which can subsequently be tracked. The tagged chromatin of hundred of cells is then combined and undergoes the ChIP-seq procedure, allowing reduction in noise associated with small input DNA.

Whilst the presence of chromatin marks has been shown to be a powerful tool to identify enhancers, localisation of key lineage-specific transcription factors has, in some cases, been shown to be a better predictor of enhancer activity [31]. The genome-wide distribution of those transcription factors is not only of particular importance for identifying their direct, downstream transcriptional targets, but it also facilitates the identification of tissue-specific enhancers. One significant advantage of using transcription factor binding is in their potential cell specificity. If the transcription factor is expressed in a cell-type specific manner, for example, only in cardiomyocytes, cell-type contamination will not affect in vivo analysis.

A large number of transcription factors have been shown to be necessary for mouse heart development [32, 33]. Moreover, human mutations for most of those factors have been suggested as the underlying cause of a variety of CHD [34]. Numerous studies have recently addressed the genome-wide distribution of such key, cardiac transcription factors (Table 1) [10, 20, 35–45]. What is striking is that comparison of results reveals wide discrepancies in the number of occupied sites identified in different studies, not only between factors but also for the same factor at different stages (Table 1). It is not yet clear whether such differences are biologically relevant, or simply reflects differences in the methodology used, the data analysis methods chosen, and differences in the precise biological model. What is clear is that a large number of parameters can affect the identification of binding regions [46].

**Table 1:** Genome-wide distribution of cardiac transcription factors

Transcription factor	Tissue	Total number of peaks	Antibody used	Biological replicates	Control	Peak calling software	GEO	References
Nkx2–5	E11.5 heart	2610	sc-8697	2	Input	MACS	GSE44576	Dupays et al. [36]
	Adult heart	6705	sc-8697	1	Input	In house	GSE35151	Van Den Boogaard et al. [10]
	Hl-1 cells	20,573	Biotinylated antibody	1	Input	Sole-search	GSE21529	He et al. [37]
	E12.5 hearts	16,899	sc-8697	1	Input	MACS	GSE70332	Ye et al. [45]
	CP	8718	sc-8697	1	ChIP in KO	In house	GSE72223	Luna-Zurita et al. [40]
	CM	25,381	sc-8697	1	ChIP in KO	In house	GSE72223	Luna-Zurita et al. [40]
	Hl-1 cells	1534	DamID	3	Input	CisGenome	GSE44902	Bouveret et al. [35]
Gata4	Adult heart	1756	sc-1237	1	Input	In house	GSE35151	Van Den Boogaard et al. [10]
	Hl-1 cells	16,753	Biotinylated antibody	1	Input	Sole-search	GSE21529	He et al. [37]
	E12.5 ventricles	43,800	Biotinylated antibody	2	Input	MACS	GSE52123	He et al. [20]
	E12.5 ventricles	11,915	sc-1237	2	Input	MACS	GSE52123	He et al. [20]
	Adult ventricles	13,504	Biotinylated antibody	2	Input	MACS	GSE52123	He et al. [20]
	CP	11,000	sc-1237	1	ChIP in KO	In house	GSE72223	Luna-Zurita et al. [40]
	CM	10,641	sc-1237	1	ChIP in KO	In house	GSE72223	Luna-Zurita et al. [40]
Tbx5	Hl-1 cells	55,872	Biotinylated antibody	1	Input	Sole-search	GSE21529	He et al. [37]
	CP	4985	sc-17866	1	ChIP in KO	In house	GSE72223	Luna-Zurita et al. [40]
	CM	8952	sc-17866	1	ChIP in KO	In house	GSE72223	Luna-Zurita et al. [40]
Tbx20	Adult heart	4012	Anti-GFP, Tbx20 GFP tagged	1	Input	QuEST	GSE29636	Shen et al. [41]
Tbx3	Adult heart	13,242	sc-17871	1	Input	In house	GSE35151	Van Den Boogaard et al. [10]
Mef2a	Hl-1 cells	1337	Biotinylated antibody	1	Input	Sole-search	GSE21529	He et al. [37]
Srf	Hl-1 cells	23,806	Biotinylated antibody	1	Input	Sole-search	GSE21529	He et al. [37]
Isl1	Adult SAN	1483	39.4D5, DSHB	1	Input	HOMER	GSE68974	Liang et al. [39]
Pitx2	12-week heart	11,280	Flag-M2	1	Input	HOMER	GSE47928	Tao et al. [42]
Hopx	E9.5 heart	3775	Flag-M2	2	Input	HOMER	GSE67251	Jain et al. [38]
COUP-TFII	E13.5 atria	2863	61214	1	IgG	MACS	GSE46498	Wu et al. [44]
Shox2	E12.5 hearts	14,271	Anti-HA	2	Input	MACS	GSE21529	Ye et al. [45]
Hey1	CM	17,874	Flag-M2	1	w/o dox	MACS	GSE60699	Weber et al. [43]
Hey2	CM	20,498	Flag-M2	1	w/o dox	MACS	GSE60699	Weber et al. [43]
p300	E11.5 heart	3597	SC-585	1	No	QuEST	GSE22549	Blow et al. [19]
	Hl-1 cells	1491	Biotinylated antibody	1	Input	Sole-search	GSE21529	He et al. [37]
	p2 hearts	6564	4771, cell signaling	1	Input	MACS	GSE32587	May et al. [21]

Many technical and methodological factors will affect both the outcome of a ChIP-seq experiment and the extent to which it can be compared with the other studies. The quality of the primary antibody used to recognise the transcription factor has been shown to be crucial, and epitope-tagging has been used to address problems caused by antibody variation and cross reactivity. This can have dramatic results on ChIP-seq results. Comparison of data obtained with an antibody against GATA4 versus a flag-tagged GATA4 shows a dramatic increase in the number of binding events (or “peaks”) from 11,915 to 43,800 [20].

The number of biological replicates can also dramatically affect the number of significant peaks identified. Pioneering studies have largely used only a single biological sample, no doubt partly due to the novelty of the technique and partly the cost of sequencing (Table 1). However, as with all biological analyses, multiple biological replicates have proved to be necessary [46]. Interestingly, increasing from two to three replicates does not appear to have significantly enhanced the quality of results [46].

The number of sequence reads has been shown to be critical for the identification of relevant peaks [47]. The number of identified binding sites increases with sequencing depth, since weaker binding sites become statistically significant with a greater number of reads [48]. Use of an appropriate control data set has also been shown to be critical [49]. This generally comprises “Input”’ DNA, (DNA prepared under the same conditions as the immunoprecipitated DNA) or an “IgG” control, (a ChIP reaction performed with an unrelated antibody). The optimum choice remains under discussion [50].

With the identification of thousands of putative enhancers, limitations enforced by the need for experimental validation of their function. Transgenic mouse reporter assays are the most commonly used approach to evaluate the function of enhancers in vivo. Such experiments consist in delivering a linear plasmid containing a reporter gene (for example, LacZ or GFP) linked to the putative enhancer into the mouse zygote, through pronuclear injection [51]. The transgenic embryos generated, generally in a transient manner, are assessed for the spatial expression of the reporter gene in the whole embryo. However, if that technique is a powerful way to identify qualitative enhancer activity, copy number, and position effect due to random transgene insertion impede reliable quantitative analysis. Moreover, mouse transgenic experiments are clearly not an appropriate technique for high-throughput testing of enhancers identified with ChIP-seq, due to their relatively high cost. Furthermore, in the context of dynamic studies, in which an enhancer has to be tested at different stages of development and/or different conditions of biological stress, this approach becomes extremely time-consuming. Stable transgenic lines would most likely need to be generated and studied in different conditions, significantly increasing the time of study as well as escalating an already high cost.

In summary, despite technological improvements which have facilitated wider adoption of the technique, design and analysis of ChIP-seq experiments remain challenging and different choices can often lead to conflicting results [52].

## Dynamic of enhancers during cardiac development

If the number of studies describing the identification of cardiac enhancers has been relatively high, the number of studies describing the dynamics of those enhancers—their differential usage between stages of development and/or in disease conditions—has been relatively poor.

Early studies established that even if evolutionary sequence conservation is a powerful tool to identify enhancers, fewer than 2 % identified in this way are active in the heart [17, 18]. Location of the transcriptional co-activator p300 proved to be to be a powerful predictor of enhancer activity in brain and limb [53] and this was, therefore, used in the mouse heart at embryonic day 11 (E11.5) [19]. Results demonstrated that most candidate cardiac enhancers identified by p300 binding are less conserved in vertebrate evolution than those in limb or brain [19].

Comparison of p300 binding between the foetal and adult human heart shows that a large proportion of the enhancers identified in this way are highly dynamic during heart development [21]. Indeed, 48 % of human cardiac enhancers active in the adult are also identified in foetal hearts, whilst only 21 % of foetal cardiac enhancers are identified with adult tissue [21]. Similar results are obtained when comparing the embryonic (E14.5) and the adult mouse heart, using the chromatin marks H3K4me1 or H3K27ac [54]. Such dynamic enhancer usage appears to reflect distinct biological function; enhancers specific to embryonic stages are associated with genes expressed during cardiac differentiation, whilst adult enhancers are associated with genes important for adult heart function [54].

Interestingly, comparison of enhancers (identified through p300 binding) between the postnatal day 2 mouse heart and the approximately equivalent 16-week-old human foetal heart shows only a 21 % overlap. This suggests a considerable and, perhaps, unexpected degree of species specificity for cardiac enhancers [21]. Since even poorly conserved human enhancers are able to drive cardiac expression in a mouse cardiac transgenic assay, even if enhancer usage between species is different, the cardiac transcription factors that regulate those enhancers are most probably the same [21].

Similarly dynamic activity of cardiac enhancers is found comparing the chromatin mark H3K27ac at different stages of mouse heart development (E11.5, E14.5, E17.5, P0, P7, P21, and P56) [22]. Indeed, only 3 % of cardiac enhancers identified in this way are predicted to be active at all stages examined. That extensive and fast turnover is illustrated with only 45 % identified actives in at most two consecutive time points [22]. The large majority of cardiac enhancers identified in this way are predicted to have a highly restricted, temporal window of activity during development, which, in turn, suggests that dynamic developmental processes are regulated by the transient activities of such enhancers.

Interestingly, substantial differences are observed in sequence conservation of putative enhancers within a given tissue across time points and also across tissues at the same timepoint [22]. As mentioned above, heart enhancers show relatively weak conservation compared with enhancers identified in either liver or more dramatically, forebrain. However, it is interesting to note that this conservation is not constant during development; enhancer conservation is maximal at early stage of heart development when compared with enhancers active in the adult heart [22].

As with heart development, enhancer usage during the differentiation of ESCs into cardiomyocytes is also highly dynamic [55]. Moreover, the distribution of four different chromatin marks at four different stages of ESC differentiation (ESC, mesoderm, cardiac progenitor, and cardiomyocytes) shows that genes with similar expression patterns can show substantial variation in chromatin states during cardiac differentiation. These results suggest that histone mark profile is not very useful for predicting dynamics of gene expression [55].

Dynamic enhancer usage is also observed when the binding of cardiac transcription factors, such as GATA4, is used to identify heart enhancers [20]. 66.5 % of the adult GATA4-bound regions are occupied by GATA4 in the E12.5 foetal heart, whilst 80.1 % of foetal GATA4 regions are not bound by GATA4 in the adult heart. Those proportions are similar to those found by assaying chromatin marks, with again, a much larger number of enhancers predicted to be active during development than in the adult. Furthermore, the authors also noted that GATA4 binding, which is mainly distal to the transcription start site (TSS) in the embryonic mouse heart, is shifted more proximal to the TSS in the adult heart.

Foetal-specific, shared, and adult-specific GATA4 regions were associated with different co-enriched-binding sites, with, for example, MEF2 and TEAD1 motifs enriched in foetal-specific regions, whilst adult regions were enriched for the EGR-1 motif. The functional consequence of such association remains undocumented.

GATA4 binding is not only changed dynamically between embryonic development and the adult heart, but also in the pathological state of cardiac hypertrophy [20], a condition in which GATA4 is well known to play a role [56]. When hypertrophy of the heart is induced by pressure overload, only 49 % of the GATA4 bound regions are found shared between ascending aortic band and the sham condition [20]. In pathological stress, such as pressure overload, reactivation of a foetal gene expression programme has been suggested to occur [57]. However, if there is clearly a redistribution of GATA4 binding under pathological stress, the redistribution to foetal-specific enhancers is relatively small. Indeed, only 20.5 % of stress-induced GATA4 binding was on foetal regions, whilst 40 % was to regions bound in neither foetal nor the adult heart [20]. These results suggest that under pathological hypertrophy, rather than reallocation to foetal enhancers, GATA4 binds to a set of stress-specific, cardiac enhancers. Similar results are found with the regulation of Nppa–Nppb gene cluster during cardiac development and hypertrophy [58]. Those two genes are induced by stress and are well-described clinical markers of heart failure. In that study, the distribution of H3K27ac and the association of Pol2 across the locus reveal that foetal expression and stress-induced Nppa expression is dependent on different enhancers, again suggesting that the foetal and stress transcriptional regulatory networks are different [58].

As observed in vivo, the binding pattern of GATA4, NKX2–5, and TBX5 is all highly dynamic during ESC differentiation into cardiomyocytes [40]. During the transition of cardiac progenitors to cardiomyocytes in vitro, more than 50 % of the binding events occur at a single, specific stage [40]. Moreover, the same study highlights the extensive co-binding of these cardiac transcription factors on common enhancers regulating cardiac gene expression. Interestingly, in a null background for NKX2–5, TBX5, or both, a significant ectopic binding of the remaining factors is observed [40]. This suggests that interdependent binding may also be necessary to prevent transcription factors from distributing ectopically and activating lineage-inappropriate genes. Such a mechanism of ectopic reallocation of transcription factor binding is also observed when the genome distribution of the transcription factor NKX2–5 is compared with that of a CHD-associated, mutated form of the protein [35]. If NKX2–5 mutants’ proteins fail to bind the majority of its wild-type targets in HL-1 cells, they are still able to recognise a large number of them along with a unique set of ectopic sites [35]. The significance of those ectopic sites of binding in the genome is not yet clear, but in the context of CHD, they could affect transcription in either a positive or dominant negative manner [35].

Extensive co-binding of NKX2–5 has also been observed with both TBX5 and SHOX2 [45]. In the E12.5 mouse heart, nearly 80 % of NKX2–5-bound regions overlap with SHOX2 binding regions [45]. Extending that co-occupancy analysis with a TBX5 data set published previously [37], the authors found that 67 % of the SHOX2–NKX2–5 co-occupied sites are also occupied by TBX5 [45]. Such significant co-occupancy of enhancers is consistent with observations indicating an antagonising role of SHOX2 on the transcriptional output of NKX2–5 in the pulmonary vein of the developing heart [45].

Our own efforts to identify active cardiac enhancers in the E11.5 mouse embryonic heart demonstrate that, at least at this stage, this is achieved more effectively using NKX2–5 binding than by p300 binding [19, 36]. 83 % of enhancers enriched for NKX2–5 binding versus 53 % for those enriched for p300 drive expression in the mouse heart. This increase in efficiency is mainly due to a reduction in the number of false positives. This suggests that binding of a critical cardiac transcription factor is an efficient predictor of cardiac enhancer activity, an observation consistent with the previous findings in equivalent studies using blood cells [31].

**Fig. 1:**
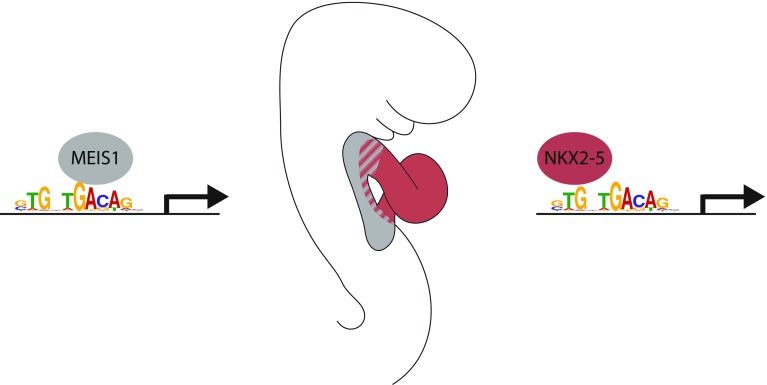
Mechanism of transcriptional regulation by successive binding of MEIS1 and NKX2–5 on cardiac enhancers. NKX2–5 and MEIS1 are able to bind in vitro on a DNA motif (GTGNTGACAG) which is an overlapping-binding site for the two transcription factors. As cardiac progenitors differentiate in the mouse embryo, they successively experience high levels of MEIS1 expression in the secondary heart field (*grey*) followed by high levels of NKX2–5 expression in the heart tube (*red*). Cardiac enhancers with an overlapping-binding site for MEIS1 and NKX2–5 are bound successively by those factors

Sequence analysis of NKX2–5-bound regions suggests that NKX2–5 and MEIS1 share an overlapping-binding site which is present in a number of cardiac enhancers in vivo (Fig. 1). Interestingly, these two transcription factors are sequentially expressed in the differentiating cardiac progenitors of the embryo. As cardiac progenitor mature, they experience successively high levels of MEIS1 in cardiac progenitors, followed by high levels of NKX2–5 in differentiated cardiomyocytes. This suggests a simple mechanism of transcriptional regulation, in which one factor successively replaces the other at cardiac enhancer harbouring the shared-binding site (Fig. 1).

The mesodermal core of the branchial arches contains a population of cardiac progenitor cells which populate the outflow tract of the developing heart [59]. Strikingly, nearly 30 % of NKX2–5-bound regions are also bound by MEIS in the first branchial arch [60], suggesting that this mechanism can be extended to an entire subset of cardiac enhancers. A similar mechanism of transcriptional regulation through an overlapping-binding site has been described for the gene *Fgf10* [61]. A cardiac enhancer of that gene is successively bound by ISL1 and NKX2–5 [61]. That *Fgf10* enhancer is activated in cardiac progenitors by high levels of ISL1 and is repressed in the myocardium by high levels of NKX2–5, which is likely to act both directly and indirectly through *Isl1* suppression [61]. Taken together, these findings suggest that overlapping transcription factor-binding sites in cardiac enhancers might be of broad significance during the differentiation of cardiac progenitors. Moreover, such a mechanism of replacement of one transcription factor by another has been found in other systems. When ESCs become specified to form neural precursors and subsequently differentiate into neurons, an ordered and sequential binding of Sox2, Sox3, and Sox11 to target enhancers drive neurogenesis. In this case, Sox2 binding would first preselect neural genes in ES cells, ensuring their proper activation in neural precursors and then inducing neuronal differentiation [62].

## Conclusion

Recent progress in ChIP-seq techniques has allowed great advances in the systemic identification of cardiac enhancers active during cardiac development, as well as in the normal and pathological adult heart. With the decrease in cost and improvement in new technologies, such as single-cell studies, identification of such enhancers in different pathological conditions as well as a more detailed spatial resolution in the mouse embryonic heart should lead to a better understanding of the gene regulatory network responsible for cardiovascular development and differentiation.
